# The Chornobyl Dogs: Why Scientific Evaluation Must Accompany Population Management

**DOI:** 10.3390/ani16192973

**Published:** 2026-09-22

**Authors:** Jean-Jacques Tuech, Olivier Guillin, Jean Pinson, Gaël Nicolas, Timothy A. Mousseau, Jennifer A. Betz

**Affiliations:** 1 Department of Digestive Surgery, Rouen University Hospital, F-76000 Rouen, France; 2 Espace Régional de Réflexion Éthique, CHU Rouen, F-76000 Rouen, France; 3 Univ Rouen Normandie, Normandie Univ, Inserm U1245 and Department of Genomic and Personalized Medicine in Cancer and Neurological Disorders, CHU Rouen, F-76000 Rouen, France; 4 Centre Hospitalier du Rouvray, Service Hospitalo-Universitaire de Psychiatrie, Centre d’Excellence Thérapeutique, F-76300 Sotteville-lès-Rouen, France; 5 Univ Rouen Normandie, Normandie Univ, Inserm U1245 and Department of Genetics and Reference Center for Developmental Disorders, CHU Rouen, F-76000 Rouen, France; 6 Department of Biological Sciences, University of South Carolina, Columbia, SC 29208, USA; 7 Clean Futures Fund, Godfrey, IL 62035, USA

**Keywords:** Chornobyl, semi-feral dogs, epigenetics, DNA methylation, intergenerational vs transgenerational inheritance, low-dose ionizing radiation, free-roaming dog population management, animal welfare, biobanking, research governance, One Health

## Abstract

Nearly four decades after the 1986 reactor explosion, several hundred semi-feral dogs persist within the Chornobyl Exclusion Zone. Genomic work has characterised their population structure and shown that radiation-induced mutation does not, on current evidence, drive the differentiation between groups; the cause of that differentiation remains unresolved, and their epigenome has never been examined. These dogs are a potentially valuable model for chronic, low-dose, multi-stressor exposure, not because they are “radiation-adapted”, a contested claim, but because they are a human-adjacent, densely sampled, pedigreed mammal for which comparative canine–human tools already exist. Since 2017, a humane sterilisation programme, carried out at the request of and in cooperation with the Ukrainian authorities, has reached near-complete coverage of the established populations. That programme was justified: dogs are formally prohibited in the zone, they injure workers, free-roaming animals inside an operating nuclear facility raise security as well as veterinary concerns, and reproduction was outpacing what the environment could support. Our argument concerns timing rather than policy. The scientific value of this population was never evaluated while it was still breeding, and the archived material that does exist, roughly 400 dogs including parent–offspring trios and repeat-sampled individuals, was assembled for population genetics rather than for the regulatory questions now worth asking. That archive should be analysed for methylation and expression now, as this is the only access to the population as it was. The animals themselves have not gone: several hundred identified dogs are still alive there, so sampling at veterinary contact and the retention of tissue at natural death or clinically indicated euthanasia remain possible, provided they are planned and funded in advance rather than improvised. More generally, the scientific evaluation of a population should accompany the decision to manage it rather than follow it, because sterilisation is not reversible at the population scale and an archive cannot be assembled retrospectively. We set out what such an evaluation requires at the outset of a programme. Its most concrete element asks nothing of the animals: a sterilisation clinic is the only routine access to a free-roaming population under anaesthesia, and the reproductive tissue it removes—the one material capable of addressing germline questions—is discarded as surgical waste.

## Simple Summary

When people were evacuated after the 1986 Chornobyl accident, the dogs left behind survived and bred, and several hundred of their descendants still live in the contaminated exclusion zone. Since 2017, a veterinary programme, working at the request of the Ukrainian authorities, has sterilised and vaccinated these dogs. That work was needed. Dogs are not officially allowed in the zone, they sometimes bite the people who work there, and they were breeding faster than the place could support, with many puppies dying young. The programme has now reached almost all of the established animals. Recent genetic studies have described these dogs, but the chemical marks that sit on top of their DNA and help control how genes are used have never been examined. Because the dogs no longer breed, the part of that question which needs puppies born to exposed parents can no longer be asked. The animals themselves are still there, however, and much can still be learned from them while they live and from the tissue that becomes available when they die of old age or are euthanised for medical reasons. Our argument is about timing rather than policy. While these dogs were still breeding, the question of what could be learned from them was raised by the scientists involved, but it was never put to the authorities who decided how the dogs would be managed, and there was no established way of doing so. By the time it could have been put, the question had answered itself. The frozen samples already collected should be analysed now. And where similar decisions are still to be taken, the scientific question should be asked at the same time as the decision to manage the animals, not years afterwards.

## 1. Introduction

On 26 April 1986, the explosion of reactor No. 4 at the Chornobyl nuclear power plant became one of the defining industrial disasters of the twentieth century [1]. At the time, the plant lay in the Ukrainian Soviet Socialist Republic, part of the USSR, and the consequences were borne across the Union: the heaviest fallout fell not only on present-day Ukraine but also on Belarus and parts of western Russia, and the response was organised at Union level [1,2]. It is important to keep this shared history in view. The event forced the evacuation of more than 120,000 people, contaminated large territories unevenly, and remains a reference point for nuclear safety worldwide.

In the prolonged absence of residents, parts of the exclusion zone have become an unplanned refuge for wildlife [3]. Among the animals that remained were the dogs left behind during the evacuation. They survived and bred, and several hundred of their descendants now live around the power plant and in the abandoned towns [4,5]. These dogs are more than a curiosity. They are a potential model for the biology of chronic environmental stress, and they raise a genuine management dilemma: how should a free-roaming population that is also of scientific interest, and that lives alongside recovering native fauna, be handled?

Why dogs, rather than the wolves, boar, lynx or Przewalski’s horses that also occupy the zone? The answer should be stated at the outset, because it is what makes this population worth arguing about. The dog is the classic sentinel species for human environmental exposure: it shares our households, our ground, much of our diet and many of our diseases, and it has long been used to anticipate human health effects of environmental contaminants [6]. This matters because the human evidence base on radiation risk is dominated by acute, high-dose cohorts such as the atomic-bomb survivors [7], whereas the exposure of interest here, and in any future contamination event, is chronic, low-dose and lifelong. Comparative canine–human genomics, immunology and oncology are mature fields [8], and dual-species methylation clocks that translate directly between dog and human already exist [9,10], so the tools needed to carry a finding across to human health are established rather than aspirational.

We do not claim that the zone’s wild species are unstudiable. The opposite is true, and the contrast is instructive. Love and colleagues fitted grey wolves in the Belarusian sector of the zone with GPS collars carrying integrated dosimeters, reconstructed individual lifetime doses, and reported altered leukocyte composition, blood-transcriptome modules whose expression tracked measured dose, and candidate immune loci showing zone-specific divergence [11]. That work is a proof of principle for the kind of functional data we advocate, and its individual, GPS-linked lifetime dosimetry is better than anything currently available for the dogs, for which the best exposure data are single-timepoint internal caesium-137 body burdens measured during clinic visits, ranging from undetectable to roughly 30,000 Bq/kg [12]. What the dogs offer is complementary rather than superior. The sampled dog population is more than an order of magnitude larger, with roughly 300 individuals genotyped, some fifteen family groups resolved and parent–offspring trios available [4], whereas the wolf transcriptomic comparison rested on nine animals [11]. The dogs are reached through the existing veterinary clinics rather than by trapping expeditions, and identified individuals have been resampled across years [4,5,13]; that resampling is opportunistic rather than systematic, since campaigns are directed at animals not yet treated and there is neither the means nor the reason to recapture a dog already sterilised and vaccinated. Tissues beyond blood can be obtained without additional harm at surgery. Their generation time is far shorter, so substantially more generations separate them from 1986 than the seven or so estimated for the zone’s wolves [11], which matters for any question about cumulative or cross-generational effects. Additionally, they live in closer and more sustained contact with the small human workforce that remains. Whether the dogs are as site-faithful as often assumed is a separate question, and one we deliberately leave open below.

We set out the scientific case carefully, distinguishing what is established from what is hypothesis. We then argue that the decision to manage such a population and the evaluation of what could be learned from it belong together in time. Chornobyl serves here as the case that demonstrates the problem rather than as a programme we ask anyone to change.

## 2. What Is Currently Known About the Chornobyl Dogs

The most detailed picture comes from population genomics. Spatola and colleagues genotyped 302 dogs and identified roughly 15 family groups distributed across three main locations: the Chornobyl Nuclear Power Plant (ChNPP) site together with the neighbouring abandoned city of Pripyat, the city of Chornobyl some 16 km away, and the satellite town of Slavutych about 45 km distant [4]. It is worth being precise about this structure, because it is often misreported. The two principal subpopulations are not “the power plant” versus “Pripyat.” Pripyat groups with the ChNPP as Population 1; the second subpopulation is Chornobyl City (Figure 1). These two form largely separate breeding groups with limited, though non-zero, gene flow, and parent–offspring links across locations indicate some movement between them [4]. A complementary study with a denser marker set from about 100 animals confirmed this structure and reported low heterozygosity, consistent with inbreeding in small, partially isolated groups [13].

These dogs are not a pristine “wild” lineage. They share ancestry with shepherd-type breeds and show evidence of recent admixture with modern purebred dogs, so the population is best understood as a semi-feral system formed by the interbreeding of abandoned domestic dogs and free-roaming animals [4]. This admixture history is itself scientifically interesting, and it must be accounted for before any locus is interpreted as environmentally shaped [4,13]. That structure is also not static, and in two directions at once. Since 2022, dogs have entered the zone from evacuated villages and from areas disrupted by the armed conflict, and some of these newcomers are described as less habituated and more aggressive than the resident animals (A. Simon, Taras Shevchenko National University of Kyiv, personal communication, 7 August 2026). At the same time, sustained sterilisation has brought the established populations to near-complete coverage, so that very few reproductively intact animals remain among them. The published genomic picture [4,13] therefore describes a population that has since been both reduced in its original component and diluted by immigration of unquantified magnitude. This is a confounder for any future analysis that treats the present-day dogs as direct descendants of the animals left behind in 1986, and it is the strongest reason to interrogate the earliest archived material, whose provenance is unambiguous, before more time passes.

On radiation specifically, the evidence is more equivocal than popular accounts suggest. Across taxa in the zone, reported biological effects have ranged from undetectable to significant depending on the species, the endpoint and the method of dose estimation [14,15], and comparable disagreements surround the wildlife literature at Fukushima [16,17]. Genome-wide scans have flagged candidate loci linked to DNA repair, immune function and oxidative-stress responses [13], yet a dedicated analysis found that the overall mutation rate in these dogs is not elevated relative to other dog populations, indicating that radiation-induced mutation does not account for the observed differentiation [18]. The 2023 studies were foundational population-genetic work and did not claim a rare evolutionary phenomenon; the 2024 analysis is not a correction of them but an extension that narrows the field of explanation. What it leaves behind is an honest gap: the driver of the genetic differentiation between these groups has not been identified. That unresolved gap is itself among the strongest arguments for adding methylome and expression data, the one layer no one has looked at. A comparable lesson comes from the zone’s wolves. Love and colleagues reported altered leukocyte composition, co-expression modules whose expression correlated with individually measured radiation dose, and candidate immune and oncogenesis-related loci showing zone-specific divergence [11]. Whatever the eventual interpretation of those signals, that study shows that regulatory and functional data can resolve structure in this system, which sequence-level scans alone did not, and the dogs remain the untouched case.

Contamination in the zone is highly heterogeneous rather than a simple gradient from the reactor: the main deposition trace ran north-west toward Belarus, and localised hotspots such as the “Red Forest” west of the reactor coexist with relatively cleaner ground nearby [2] (Figure 1). Any biological signal must therefore be separated from a range of co-occurring stressors, including heavy metals and other chemical pollutants, variable nutrition and human feeding by the remaining workforce, parasites and infectious disease, and the demographic effects of a small, admixed population [19,20]. We return to these confounders throughout.

**Figure 1:**
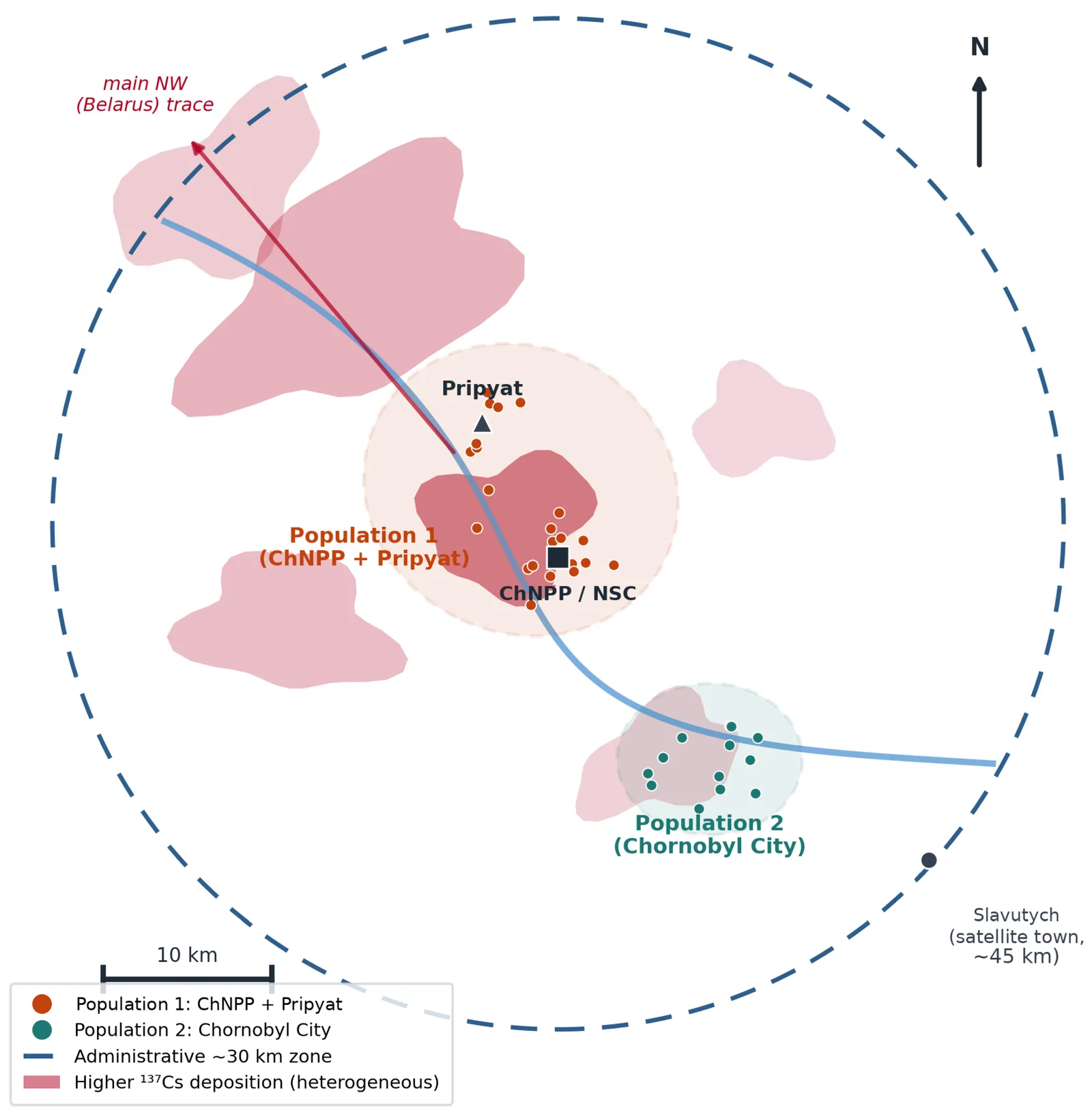
Schematic of the Chornobyl Exclusion Zone showing the two principal dog subpopulations and the heterogeneous pattern of contamination. Following Spatola et al. [4] and Dillon et al. [13,18], Population 1 comprises dogs at the ChNPP site together with the adjacent abandoned city of Pripyat (orange), and Population 2 comprises dogs of Chornobyl City (teal), about 15 km away; roughly 15 family groups are nested within this structure, and further animals occur at checkpoints and in Slavutych (~45 km). We do not label group sizes, because reliable per-group counts are not available: manual surveys suggested approaching 1000 dogs near the power plant in spring 2017, and the source states that accurate population estimates do not exist [12]. Contamination is spatially heterogeneous rather than radial: the main ^137^Cs deposition trace ran north-west toward Belarus, with localised hotspots near the reactor (schematic after published caesium-deposition maps [2]). Positions are approximate and not to exact scale.

## 3. From Genomes to Epigenomes: An Unexplored Dimension

Organisms do not respond to chronic stress only by changing their DNA sequence; they also change how that sequence is regulated. This regulatory layer, the epigenome, comprises DNA methylation, histone and chromatin modifications, and non-coding RNAs. These marks are environment-responsive and can change within a lifetime [21]. In the Chornobyl dogs, the genome has begun to be studied, but the epigenome has not been characterised at all. That is the gap we wish to highlight.

Ionizing radiation is known to act on this layer. Controlled and field studies report altered DNA methylation, histone changes and shifts in non-coding RNAs after low-dose or chronic exposure, with effects on DNA-repair and stress-response pathways [21]. Genome-wide hypermethylation was reported decades ago in Chornobyl pines, and comparable responses have been described in other plants and in aquatic and terrestrial animals along contamination gradients [21,22]. Importantly, however, differential methylation is not evidence of adaptation. Age, inflammation, infection and parasite load, diet, and differences in the cellular composition of a sample can all shift methylation, and these must be excluded before any epigenetic difference is read as an adaptive or radiation-specific signal [23]. We do not assume a distinctive “Chornobyl signature” exists. Whether any exposure-associated epigenetic pattern is present in these dogs is an open question, and the most likely alternative explanation is not noise but genetics: the two subpopulations differ in ancestry and admixture [4,13], and much variation in DNA methylation is under genetic control, so between-group methylation differences could simply track between-group genetic differences. Demonstrating an exposure-associated signature would therefore require a design integrating genomic, environmental, clinical and cell-composition data on the same animals, rather than a methylation comparison alone.

The inheritance of such marks requires careful language, because the field is often over-stated. It is standard to distinguish *intergenerational* effects, in which the exposed parent and the directly exposed germline (and, for a pregnant female, the in utero offspring and its germline) carry the mark, from truly *transgenerational* inheritance, which is demonstrated only when a mark persists in generations never exposed to the trigger, that is, the third generation and beyond (F3+) for maternal exposure. The threshold is set there because an exposed pregnant female exposes three generations at once: herself, the fetus she carries, and the germ cells already forming within that fetus, which will give rise to the following generation. F3 is therefore the first generation that is never itself exposed [24]. Transgenerational epigenetic inheritance is well established in plants and some invertebrates [25], but its existence and significance in mammals remain contested, largely because of the extensive epigenetic reprogramming that occurs in the germline and early embryo [24,26]. The most directly relevant evidence is discouraging. A whole-genome study of father–mother–child trios among the children of Chornobyl clean-up workers found no association between parental radiation exposure and germline de novo mutation [27], and a survey of nematodes living in the zone found no systematic effect of environmental radiation on their genomes [28]. Neither study examined methylation, which is our point, but both counsel strongly against expecting a large transgenerational signal here. Experimental demonstrations, such as differential methylation persisting to the F3 generation in irradiated zebrafish [29], are therefore best read as proofs of principle in tractable models, not as evidence that the phenomenon occurs in dogs. We treat transgenerational inheritance in the Chornobyl dogs strictly as an open hypothesis, and we add a design constraint that is easily overlooked: in a population that remains exposed, a multi-generation study cannot by itself distinguish genuine epigenetic inheritance from the repeated induction of similar marks in each generation. A design extending to F3 and beyond is therefore necessary but not sufficient; a valid test would additionally require descendants that are no longer directly exposed, together with genetic and environmental controls. This makes the question a distant one, and we raise it to mark the boundary of what could eventually be asked rather than to propose it as an early objective. The nearer-term, testable questions concern within-lifetime and intergenerational effects.

A fair objection is that the acute phase of contamination ended decades ago and that ambient exposure is now comparatively stable, so it is reasonable to ask what a longitudinal design would actually be investigating. It is three things, none of them the ambient dose rate itself: First, the individual: methylation age, telomere dynamics and the microbiome change across a lifetime, and the question is whether they change faster, or differently, in animals carrying higher cumulative burdens. Second, the generational transfer: whether marks present in a parent appear in offspring conceived and raised under measured exposure, which can only be observed as litters are actually born. Third, the environment, which is less stable than it appears: the 2022 conflict, the associated fires, interruptions to feeding and veterinary provision, and any future change in zone management are perturbations whose biological consequences cannot be inferred from samples banked before they occurred. The questions that require living animals are therefore about trajectories and transmission, not about re-measuring a static field. At Chornobyl, only the second of these three now lies beyond reach, and Section 5 and Section 6 explain why and what follows from it.

Several of these questions are tractable with existing tools, provided their limitations are acknowledged. DNA-methylation “clocks” estimate biological age and have been developed for dogs and, more recently, across mammalian tissues [9,10,30]. They are powerful but not turnkey: their readings are tissue-specific, sensitive to cell-type composition, and confounded by inflammation, disease, metabolism and other lifestyle factors; what they track biologically is still being resolved [23]. With adequate design and controls, the following could be examined in this population, and in few others:

**Epigenetic age acceleration.** Whether dogs in more contaminated settings show accelerated methylation age relative to chronological age, after adjusting for tissue, cell composition, inflammation and disease [9,10,23,30].
**Differential methylation and expression at stress pathways.** Whether the repair, oxidative-stress and immune loci flagged by genomic work [13] are differentially regulated, distinguishing environmental effects from admixture and from confounders [21,23], as transcriptomic work in the zone’s wolves suggests is feasible [11].
**Intergenerational effects and a framework for testing transgenerational inheritance.** Whether marks associated with the zone appear in directly exposed offspring (intergenerational), with any claim of transgenerational persistence reserved for an explicit F3+ design [24,26,29].
**Companion sub-genomic markers.** Telomere dynamics [31], mitochondrial integrity [19] and the gut and skin microbiome [32], which together form a dynamic phenotype relevant to both animal and human health.

Work on living animals bears directly on welfare; therefore, what the sampling would actually involve should be first set out. Blood is the primary and most accessible tissue and is sufficient for methylation, transcriptomic, telomere and mitochondrial assays; buccal swabs and hair provide low-burden alternatives; faecal swabs capture the microbiome. Routine biopsies of healthy free-roaming dogs are not in question. The one setting in which multiple tissues can be obtained without additional harm is the sterilisation clinic itself, where gonadal tissue is removed as part of the procedure itself, and where post-mortem material is occasionally available. This matters scientifically as well as ethically, because methylation is strongly tissue-specific [10,23]: a blood-based study answers some questions and not others, and cross-tissue claims require exactly the surgical and post-mortem material the clinics can supply. Sampling and welfare provision are therefore not in tension here; they run through the same door. Section 6 returns to this point, because it is the practical heart of what we propose.

One caveat deserves emphasis. It has been suggested that a sedentary lifestyle would make these dogs ideal sentinels of a fixed local exposure. Site fidelity is plausible given the largely separate breeding groups [4], but individual movement has not been quantified, and workers in the zone report anecdotally that dogs do move, at least between the ChNPP and Chornobyl City. Parent–offspring links across locations are consistent with this [4]. Establishing individual exposure histories would require GPS tracking or mark–recapture [5]. We therefore treat exposure stability as a hypothesis to be measured, not an assumption, and any dosimetric claim should await it.

## 4. Archived Samples and Living Populations: Complementary, Not Interchangeable

An obvious objection is that the value we describe could simply be banked: collect DNA, tissue, gametes and cell lines, cryopreserve them, and let welfare-driven sterilisation proceed. Much of that objection holds. Properly collected and stored frozen blood and tissue are perfectly suitable for recovering DNA methylation; frozen material also yields transcriptomic and non-coding-RNA snapshots, mitochondrial data, and, from a faecal swab taken at the time of sampling, a microbiome profile for that moment. None of these requires a living animal at the time of analysis (Figure 2).

The point extends further, and in a direction that strengthens rather than weakens the field. Substantial archives already exist. Around 400 dogs have been sampled, family groups and parent–offspring trios are resolved, and some individuals have been sampled repeatedly across years [4,13,33]; whole-genome and marker data from that work are publicly available [4,13,18]. These collections are not a single time point, and they predate both the bulk of the sterilisation effort and the arrivals of the past few years, which makes them a baseline that can no longer be reconstructed. Around 300 of these animals form the genotyped cohort on which the published population-genetic work rests [4,13]; further material was collected in later campaigns, including the New Safe Confinement campaign of 2022, and its extent and condition are not established. An epigenomic and transcriptomic analysis of banked Chornobyl dog material would be among the most informative studies that could be undertaken today, and it requires no new animals and no change to any policy.

What archives cannot supply is the future. They are finite, retrospective, and were assembled for population genetics rather than for epigenomic work, which matters because storage adequate for genotyping is not always adequate for methylation or RNA. A frozen collection cannot generate new longitudinal series on animals sampled forward in time; it cannot record transmission from parents to offspring under continuing, measured exposure; it cannot support a purpose-built design extending to the third generation; it cannot capture how a population responds to conditions that have not yet occurred; and it cannot link molecular marks to health outcomes and lifespan that have not yet happened (Figure 2). It matters, however, to be precise about which of these a sterilised population forecloses, because the answer is narrower than it first appears. What sterilisation ends is transmission: no offspring will be conceived and raised under measured exposure, and no design extending to the third generation is now possible at Chornobyl. What it does not end is the population. Several hundred identified animals are still alive there, of known identity and usual location, with individual exposure data available for some, and they are still ageing. Blood, buccal and faecal sampling remain possible whenever an animal is handled, whether for veterinary care or under a purpose-designed protocol; longitudinal series on individuals sampled forward in time therefore remain available, as does the linkage of molecular marks to the health outcomes and lifespans that these animals are living out now. Of the three things the paper has treated as lost, only the first is genuinely gone.

**Figure 2:**
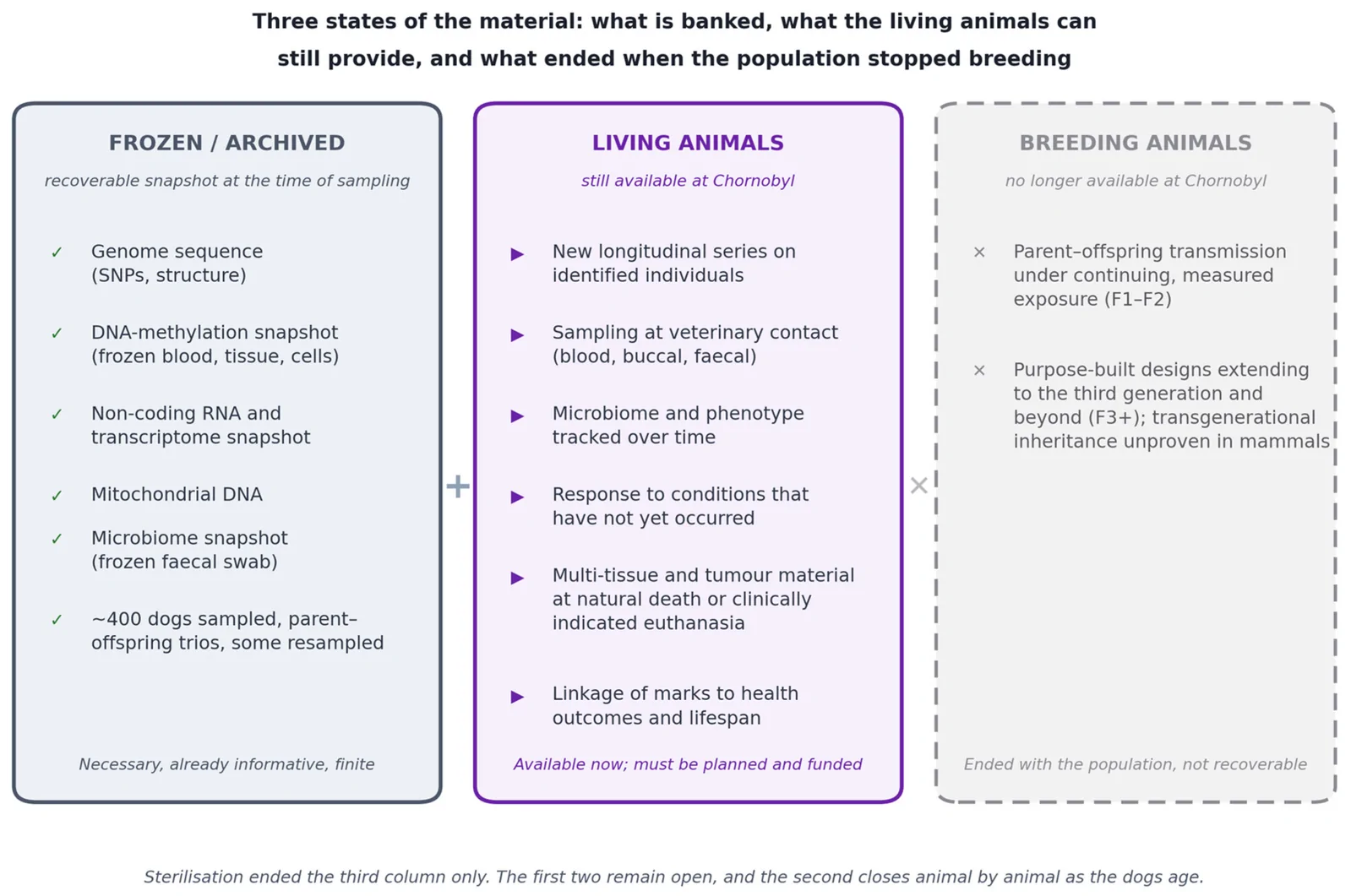
What archived samples and the living animals each provide. Properly stored frozen material recovers the genome and a snapshot, at the time of sampling, of DNA methylation, transcriptome and non-coding RNAs, mitochondrial DNA and the microbiome (from a frozen faecal swab); existing collections already include roughly 400 dogs, parent–offspring trios and some individuals resampled over several years [4,13]. These archives are necessary and should be analysed first, but they are retrospective and finite. Prospective data require living animals, but not all of it requires breeding ones, and the distinction matters at Chornobyl. New longitudinal series, sampling at veterinary contact, multi-tissue and tumour material at death, and the linkage of marks to health outcomes and lifespan need only that identified animals remain alive. Parent–offspring transmission under continuing measured exposure, and purpose-built designs extending to the third generation (transgenerational inheritance being unproven in mammals [24,26]), need a breeding population and are no longer available there. The three states are complementary; none substitutes for another.

## 5. Sterilisation, Welfare and Population Management: What Was Decided and Why

Since 2017, Clean Futures Fund, a nonprofit organisation, has run annual clinics in the zone to sterilise, vaccinate and provide veterinary care to the dogs [5,12,33]. It is the only organisation providing this work inside the zone, operating under a memorandum of understanding with the State Agency of Ukraine on Exclusion Zone Management, which includes cooperation with the Chornobyl Nuclear Power Plant (ChNPP). Several bodies support the programme financially or in kind and are often named alongside it, which has given rise to a common but mistaken impression that a number of non-governmental organisations operate on the ground. Population figures deserve care, because they are weaker than is often assumed. Manual surveys conducted by the programme suggested approaching 1000 dogs in the immediate vicinity of the power plant in spring 2017, and the report of that work states plainly that accurate population estimates are not available [12]. Figures drawn from individual research studies, such as the 36 animals sampled in 2018 [12], describe that study and not the annual activity of the programme, and should not be read as programme totals. It is essential to state what these clinics are and why they exist. They were introduced explicitly as a humane alternative to culling, at a time when the removal of feral dogs had political momentum in Ukraine, and their aims—reducing rabies risk to the workforce and to visitors, decreasing population size, and relieving suffering—are legitimate [4,5]. Zone dogs face harsh winters and parasite and pathogen burdens. Historically, many died at relatively young ages from infectious disease, trauma, fighting, exposure and other consequences of the harsh environment [5,20]. Survival has since improved substantially with sustained sterilisation, vaccination, feeding and veterinary care, and many animals in the established population are now more than ten years old.

Conditions on the ground are not apparent from the published literature, and they matter here. Dogs are formally prohibited both within the exclusion zone and on the power-plant site itself; the population persists not because any policy permits it but because people feed and tolerate the animals. The dogs also cause tangible harm: between five and ten people were bitten in the spring of 2026, and free-roaming animals inside an operating nuclear facility raise physical-security as well as veterinary concerns. Against that background, the programme works towards sterilisation of the great majority of the population, with workers in the zone reporting any remaining intact animals so that a subsequent mission can reach them; the sterilised animals are then left to live out their lives, and culling is not contemplated. Those working in the zone regard this combination as the best available outcome for the dogs (A. Simon, Taras Shevchenko National University of Kyiv, personal communication, 7 August 2026). The programme has been interrupted more than once. A campaign planned for the New Safe Confinement in May 2022 was cancelled by the invasion of that February; an assessment visit followed in June, during which areas were identified that could be sectioned off into corrals so that dogs could be conditioned to enter them, and a full campaign was carried out in October 2022.

Two further considerations strengthen the case for management. The first is zoonotic. Ixodid ticks in Ukraine carry Borrelia, Anaplasma, Rickettsia and Babesia [34]; Borrelia and other zoonotic agents have been documented directly in ticks collected inside the exclusion zone [35]; and pathogen prevalence has now been compared between ticks and dogs at Chornobyl itself [20]. Nationally, reported Lyme borreliosis incidence rose from 0.12 to 12.8 cases per 100,000 between 2000 and 2018, and the central region, which contains the Kyiv, Zhytomyr and Chernihiv oblasts that hold and border the zone, has been consistently among the most affected [36]. The authors of that survey caution that improved awareness and diagnostic capacity account for part of the rise, so the trend should be read as directional rather than exact. The mechanism nonetheless matters here: vector-borne pathogens are difficult to control in a free-roaming population because, although antiparasitic preventatives are administered to many animals through the veterinary programme, repeated treatment cannot be delivered reliably to every dog at the required intervals, so a larger dog population means a larger reservoir and greater risk to the workforce and to native fauna. The second is ecological. These animals are not native wildlife. Free-roaming domestic dogs are among the most damaging introduced predators globally, implicated in vertebrate extinctions and threatening well over a hundred species through predation, disturbance, competition, hybridisation with wild canids and disease transmission [37,38]. The exclusion zone has become a de facto reserve for wolves, lynx, boar, bison and Przewalski’s horses [3]. Any proposal to sustain a breeding dog population inside it must therefore answer to conservation, not only to welfare, and hybridisation with the zone’s wolves is a specific risk that would require monitoring.

One possibility that might be thought to cut against the programme should be set aside, because it is intuitive and, on the available information, wrong. It could be supposed that capture is non-random, that the most approachable dogs are preferentially caught, and that a decade of clinics has therefore selected against tameness. Field practice does not support this. Capture teams work towards taking every animal within a targeted population, and animals that cannot be handled safely are chemically immobilised by remote darting, so that capture does not depend on how approachable a dog is; where darting proves counterproductive, as in the high-security area of the plant where a single darted animal scatters the rest, corrals have been built instead (A. Simon, personal communication, 7 August 2026; [33]). Clinic-imposed selection on tameness is therefore not a plausible driver of change in this population. The residual caveat is narrower and worth recording only as a sampling limitation: an animal that consistently evades capture is by definition absent from both the clinic record and the archive, so neither is a strictly random sample of the population.

The welfare and management literature constrains what can be claimed on either side. In free-roaming dog systems, culling typically produces only a temporary reduction, with numbers rebounding once effort ceases, whereas neuter-and-return programmes tend to be more effective over the long term and to score better on welfare [39,40]. The clearest demonstration comes from a directly comparable population: genomic reconstruction of the free-roaming dogs of the Galapagos showed that the culling campaign begun in the 1980s neither controlled population size over the long term nor reduced genetic diversity, and that the population instead increased and expanded while acquiring the genomic signatures of modern purebred dogs that were illegally imported [41]. That study also illustrates the wider point about archives, since it depended on historical samples collected decades earlier. Vaccination coverage of about 70% is the recognised threshold for interrupting rabies transmission [39,42]. Sterilisation coverage targets depend heavily on local turnover, and immigration from surrounding areas can offset local sterilisation [39,40]; this is not hypothetical at Chornobyl, since the entry of dogs from evacuated villages since 2022 (Section 2) means the managed population is open rather than closed, which raises the coverage needed to hold numbers stable. Two implications follow. First, disease control and reproductive control are not the same lever, and vaccination without reproductive management leaves the population problem unresolved. Second, numerical stability is not evidence that reproduction was under control. Before the clinics began, the dogs were breeding heavily, and high reproduction was offset by high mortality: litter after litter of puppies died of disease, malnutrition, exposure and trauma [33]. A population that appears stable on that basis has a welfare problem rather than none. Provisioning compounded it, since feeding improved body condition and better-conditioned animals bred more readily [33], so the care given to these dogs and the growth of their numbers were two aspects of the same thing.

The programme has now brought the established populations to near-complete coverage, and very few reproductively intact animals remain among them. This forecloses the possibility of a breeding cohort at Chornobyl. Whatever might once have been said for retaining a defined, well-managed group of reproducing animals for study, the population that would have supplied it has substantially ceased to breed, and what remains reproductively active belongs largely to new dogs that entered the zone after 2022 and therefore represents the dilution of the lineage rather than the lineage itself. The proposal is moot, and we do not pursue it. It should be added that a proposal of that kind carried costs of its own that were never trivial: maintaining only a subset of animals as breeders reduces effective population size and can accelerate inbreeding in a population already showing low heterozygosity [13], which is both a genetic and an animal-welfare problem [43,44]; a marginal subsistence in a stressful disaster site is not in itself a humane aspiration. What remains, and what the rest of this paper is about, is a question of sequence rather than of policy: the scientific value of this population was never assessed at a time when the assessment could have made a difference.

## 6. What Should Be Decided at the Outset

Everything above describes a decision that was never systematically contemplated. It was not, however, a decision that no one had thought of. The possibility of leaving a reproductively intact subset of the dogs for scientific study was considered by the programme’s scientific lead at the time the clinics began, for substantially the reasons set out in this paper, but it did not go beyond a thought experiment. The reasons it did not are worth stating, because they are not reasons of oversight.

The sterilisation programme was carried out within management requirements established by the Ukrainian authorities responsible for the Exclusion Zone, at their request, and maintaining a breeding population for future research was not among the objectives it was given. In 2017, the programme’s own continuation was uncertain, funding was reliant on private donations, and fewer than half the dogs had been sterilised, so that preserving a subset was not yet a relevant question: intact animals plainly remained. There was, in addition, no practical route by which such a subpopulation could have been established and maintained, and no obvious source of funding for it.

That combination is the phenomenon this paper is about. The question existed on the scientific side of the programme, where nobody was in a position to decide it; the decision sat with authorities whose mandate did not include it and to whom it was never formally put. And at no single moment did the window appear to be closing. Each campaign was a reasonable increment, each left animals still breeding, and the outcome was irreversible only in aggregate. No moment announced itself as the last one. This is why the evaluation must be scheduled deliberately at the outset of a programme rather than left to arise when someone notices: not because anyone is negligent, but because the point of no return is invisible from inside the sequence that produces it.

What follows is what should accompany the launch of a humane management programme in a population of potential scientific interest. The list is deliberately modest. Almost none of it requires that any animal be left reproductively intact, and almost none of it constrains the veterinary objective. Its cost is not always trivial, as Section 6.2 and Section 6.3 describe, but it is modest relative to the irreversibility of what is otherwise lost, because sterilisation cannot be reversed at the scale of a population and an archive cannot be constructed retrospectively.

### 6.1. Pose the Scientific Question Where It Can Be Answered

Most free-roaming dog populations will not address the question. A few, those in contaminated landscapes, those with documented and unusual ancestry, and those under a measurable and sustained exposure, plainly will. Where the question does arise, it needs a route to a decision.

The Chornobyl case shows what happens without one. The question was asked by a competent person at the right time, but had nowhere to go: it was not within the mission of the veterinary organisation, whose responsibility was welfare and management under an agreed mandate, and it was never formally put to the authority that could have mandated such an action. What is needed is not more thought but a conduit for action: an explicit discussion, at the outset, between the scientific advisers, the implementing organisation and the responsible authority, producing a recorded answer. The absence of such a conduit is not a failure of anyone within it. One mechanism deserves particular mention, because it already exists and costs nothing. Where the practitioners who do the work are included as authors on the scientific output of a programme, rather than acknowledged at the end of it, the incentive to participate changes and so does the standing of the scientific question inside the organisation. Distrust of research among practitioners is not irrational and is often the product of having supplied the material for work in which they had no part. Authorship is a poor substitute for a governance mechanism, but it is one of the few arrangements that reliably brings the two sides of a programme into the same conversation.

### 6.2. Bank What the Surgery Already Removes

This is the recommendation we would most like to see acted upon, and it is the one that costs least.

A sterilisation clinic is the best access point to a free-roaming population that will ever exist. The animal is caught, individually identified, anaesthetised and operated on. Blood can be drawn without additional harm, and buccal and faecal swabs are trivial. None of this requires a second capture, a second anaesthesia, or any procedure the animal would not otherwise undergo. The Chornobyl programme demonstrated this in part: it is the reason an archive of blood, tissue and microbiome samples exists at all [4,13].

The reproductive tissue removed at surgery deserves separate mention, because it bears on the hardest question in this field. The distinction between intergenerational effects and true transgenerational inheritance turns on the separation of germ and somatic cells in the early embryo and is particularly difficult to establish in mammals because of the reprogramming that occurs in the germline and early embryo [24,26]. Blood cannot address it: DNA methylation is strongly tissue-specific [10,23], and no blood-based study, however large, will say anything directly about germ cells. The only route is gonadal tissue, and in a free-roaming population, the only routine access to gonadal tissue is the sterilisation surgery. To our knowledge, germline methylation has never been measured in any mammal from the zone.

What such material could support is narrower than it first appears and needs to be stated exactly. Ovarian and uterine tissue, removed at every ovariohysterectomy, is the maternal side of the question, and maternal exposure is the reference case for the third-generation (F3) criterion described in Section 3 [24]; oocytes are difficult to isolate from a whole ovary under field conditions, but the tissue is unambiguously available. Male sterilisation in dogs almost always employs castration rather than vasectomy, so epididymal and testicular spermatozoa are the more tractable germline substrate: a near-pure cell population and the standard material for germline methylation work. Gonadal histology requires no molecular work at all: spermatogonia are among the most radiosensitive cell types known, although mature spermatozoa are by contrast highly radioresistant, and follicular depletion or disrupted spermatogenesis are long-established endpoints needing only fixation and a slide. Two limits must be stated against this. A whole gonad is mostly somatic tissue, comprising stroma, granulosa, Sertoli and Leydig cells, so bulk methylation does not measure the germline without cell sorting or single-cell resolution [23]. In a population that no longer breeds, such material yields the state of the germline, not the fact of transmission. The question it answers is whether exposure marks the germline differently from the somatic tissues of the same animal. That is a genuine question in its own right, and one that has to be settled before transmission can sensibly be investigated.

The decisive feature of this material is that retaining it asks nothing of the animal. The tissue is already excised, from an identified, anaesthetised animal of known usual location, and is then discarded as surgical waste. That is standard practice, and there has never been a reason for it to be otherwise. The criterion that governs what a welfare clinic can reasonably be asked to do is, in the experience of one of us (J.A.B.) as veterinary director of the Chornobyl programme, the following: where it is possible and useful, sampling will be conducted, provided it does not distress the animal or unnecessarily prolong the anaesthesia. Excised gonadal tissue satisfies that criterion completely, which is precisely what distinguishes it from an additional biopsy that a welfare clinic would rightly refuse. We propose nothing that fails this test.

What prevents it in practice is worth setting out, because it determines what the recommendation should be. Part of the obstacle is material. At Chornobyl, some gonadal tissue was collected but could not be preserved for want of freezers, preservative, back-up generators and people available to take the samples, and single-cell sequencing of sperm was an explicit intention that funding and technical constraints made impossible; freezer capacity on site is committed to primary material, and the journey out to a receiving laboratory is long enough to make the preservation of secondary samples impractical. The same constraint is visible throughout a programme that operates on donated funds and volunteer labour, and that in a single campaign stretched an anaesthetic budget calculated for a hundred animals across a hundred and twenty-five [33]. Routine sample collection was discontinued after 2024, when a smaller field team made it increasingly difficult to manage without slowing the clinical work.

The larger part of the obstacle, however, is institutional, and it applies well beyond Chornobyl. Organisations that do this work without charge are rare. Many sterilisation campaigns are run commercially, and their operators are paid to sterilise rather than to collect samples, so that sampling costs them time and returns them nothing. Welfare organisations are also understandably cautious, for reasons that deserve respect, about anything that might be perceived by donors or by the public as experimentation on animals, particularly where their primary mission is animal welfare. This account reflects the experience of one of us (J.A.B.) across high-volume sterilisation campaigns in several settings and is offered as an observation rather than as a claim about every welfare or sterilisation organisation. It follows that the Chornobyl programme, which permitted sampling throughout, is the exception rather than the model, and that an appeal to goodwill is not a plan. What is needed is that the question be settled with the implementing organisation at the design stage, on terms that respect the criterion above, and that sampling and storage be funded as a line of their own rather than assumed as a by-product of the surgery. Two kinds of cost should be distinguished here, because the paper has no interest in conflating them. The cost to the animal of retaining tissue already excised is nil, and that is what makes the proposal admissible to a welfare clinic at all. The cost to the programme is real, and the preceding paragraph describes what it has prevented. Our claim is only that it is modest relative to the irreversibility of the loss, and that it should therefore be budgeted at the outset rather than discovered afterwards.

### 6.3. Keep Sampling the Animals That Remain, and Plan for the End of Their Lives

A sterilised population is not a closed one. At Chornobyl, several hundred identified animals are still alive, and every occasion on which one of them is handled for veterinary care is an opportunity for blood, buccal and faecal sampling at no cost to the animal, exactly during the sterilisation surgery. A purpose-designed protocol could go further: these are animals of known identity and usual location, for some of which individual exposure data exist, and following them forward remains entirely possible.

The end of their lives deserves separate treatment and requires a boundary to be drawn explicitly. These dogs are ageing, and a proportion will develop the diseases of old age; euthanasia has already been necessary in individual animals because of advanced cancer and of severe arthritis resulting in loss of mobility and an unacceptable quality of life. The decision to euthanise is a clinical one. It belongs to the attending veterinarian and rests on the welfare of that animal alone, and no scientific consideration enters it or ever should. Our premise is that no animal should be euthanised, or have that decision brought forward by so much as a day, for research purposes. What we propose concerns only what becomes of the tissue after a decision taken for entirely other reasons, and the same principle would apply if animals were found dead of natural causes.

On that basis, the opportunity is considerable, and it is the one part of the prospective programme that sterilisation has not foreclosed. Post-mortem material is the only route to the multiple tissues that cross-tissue methylation questions require, since methylation is strongly tissue-specific, and a blood-based study can answer only some of them. Tumours in a species that is the classic comparative model for human cancer are of obvious interest in a chronically exposed population. And organ material linked to a documented life history is the kind of endpoint an archive assembled for population genetics cannot supply. Two practical limits should be stated: post-mortem interval degrades RNA considerably faster than DNA, so material intended for transcriptomic work needs to be stabilised quickly, and euthanasia agents themselves alter some tissue parameters. Neither is an obstacle to a protocol designed with them in mind.

This requires no change to the clinical decision-making of the veterinary programme, but it does require additional planning for collection, preservation, storage and transfer: a standing agreement about what is retained, in what form and by whom, made in advance rather than improvised at the moment an animal dies, together with the freezer capacity and the route out of the zone discussed above. Without such an agreement, the material is lost by default, because the decision has to be taken in the space of an hour by someone whose proper attention is elsewhere.

### 6.4. Record the Management Itself as a Covariate

Which animals were sterilised, when, where, by what capture method, and on what basis; together with provisioning, clinical findings, body condition and individual identity should be documented. A programme that keeps these records converts its own intervention from an unrecorded confounder into measurable metadata, and programmes of this kind already generate most of them operationally [39,45].

Provisioning deserves particular emphasis, because at Chornobyl, it was not a background variable but a demographic driver: feeding improved the dogs’ body condition, and better condition increased their breeding [33]. The welfare provision and the population problem were the same phenomenon, and the intervention was shaping the population it was measuring in more ways than sterilisation alone. Any study that treats provisioning merely as a nutritional confounder has misunderstood the system. Management is never external to the population under study, and the only defence is to record it.

### 6.5. Put the Exemption Question on the Record, and Expect the Answer to Be No

Whether any subset of animals should be left reproductively intact for research is not a question for scientists alone to settle. It belongs to the national authority, to the communities and workers who live with the animals, and to the veterinary professionals responsible for them. The argument here is only that it should be asked explicitly, at the start, of the body that can actually answer it, and that the answer should be recorded with its reasons.

We should say what the answer would probably have been at Chornobyl. Dogs are formally prohibited both within the Exclusion Zone and on the power-plant site; people are bitten; free-roaming animals inside an operating nuclear facility raise physical security as well as veterinary concerns; and free-ranging dogs would not be tolerated at an operating nuclear station in most countries. There is also the welfare argument against the proposal noted in Section 5. On the evidence available, an exemption would very probably have been refused, and we would not now argue that the refusal would have been wrong.

This does not weaken the recommendation. A question asked and answered negatively is a decision; a question never asked is not. The first leaves a record of what was weighed and can be revisited when conditions change; the second forecloses silently.

### 6.6. Define Access to the Archive Before the Archive Exists

Who may request material, on what terms, with what obligations of attribution and data return, and who decides should be defined. Programmes that bank material without an access policy tend to discover, years later, that the material is functionally unreachable: the custodian has moved on, the consent basis is unclear, or the request has no addressee. The Chornobyl material is better placed than most, since the whole-genome and marker data are publicly available [4,13,18], and the physical samples are held by identifiable institutions, but this was the by-product of the researchers involved rather than of any stated policy.

### 6.7. Stabilise and Remove the Material, and Do It Early

A population changes for reasons unconnected to any management programme. At Chornobyl, new dogs have entered the zone since 2022 from evacuated villages and areas disrupted by the conflict, while the established populations have been progressively sterilised (Section 2). The archived material now has a value that was not anticipated when it was collected, because much of it predates both processes; that was fortunate rather than planned.

The corollary is that banking is not the same as archiving. A quantity of frozen tissue held in a laboratory within the zone was lost during the power failures that followed the 2022 invasion before it could be properly archived and moved out. Material that has been collected but not stabilised, catalogued and physically removed to a secure repository amounts to an intention rather than an archive. In settings of political or infrastructural instability, which is to say in most of the settings where this kind of work is done, the interval between collection and secure deposit is itself a risk to be managed.

### 6.8. Where This Can Still Be Applied

The decisions taken at Chornobyl are not reopened by anything above, and Section 6.3 aside, the recommendations are addressed to situations in which such decisions have not yet been taken.

Free-breeding and village dog populations exist at other contaminated sites, including eastern Kazakhstan and the Marshall Islands, where population control is not currently applied and where research programmes are active. The Chornobyl situation is not universal: after the Fukushima evacuation, most pet dogs left with their owners or were gathered into shelters for later collection, and no free-roaming dog population established itself, although abandoned livestock raised comparable questions of their own. That contrast is itself instructive, since it shows how contingent the Chornobyl opportunity was. Comparable situations will arise again after any future contamination event in which domestic animals are left behind, and they arise more broadly still: humane sterilisation programmes operate in free-roaming dog populations worldwide under established welfare standards [39,45], in populations of interest to conservation genetics and to the study of domestication as well as to environmental health. In none of these settings is the cost of a tissue-banking protocol, an access policy and a recorded decision large relative to the irreversibility of omitting them.

### 6.9. What This Would Have Changed at Chornobyl

Probably not the sterilisation programme. On the evidence now available, it would have proceeded much as it did, and would have been right to do so. What it would have changed is the archive: material banked to a standard that supports regulatory as well as sequence-level analysis, reproductive and multi-tissue samples retained from surgery rather than discarded, provisioning recorded as the demographic variable it was, a stated route by which the material could be reached, and samples stabilised and removed before they could be lost to a power failure. The population would have ceased to breed in the same way and on the same schedule, but we would know considerably more about it.

This is a critique of an absent mechanism and not of the people who worked within its absence. Whatever scientific record exists for these animals exists because a welfare organisation went to the zone at the request of the Ukrainian authorities and did difficult work there under conditions of disaster and war, funding scarcity and personal risk and because the scientific work already under way in the zone recognised the opportunity and improvised a sampling programme around the clinics, acquiring as much information as possible without interfering with the primary welfare and population-management objectives. Without those clinics, there would be no sampled population, no resolved pedigrees, no genetic sequences and no archive to argue about. Chornobyl is not the case in which something was missed. It is the only case in which any of this was demonstrated at all, and that is why it warrants close examination.

## 7. Limitations

This is an opinion piece, and its argument has clear limits. No epigenomic data yet exist for these dogs, so the central proposition, that an informative epigenetic dimension is present and recoverable, is a well-motivated expectation rather than a demonstrated result. Much of the supporting evidence comes from other species, including zebrafish, rodents, plants and, within the zone itself, wolves [11,21,22,29]; interspecific extrapolation to dogs is uncertain. Transgenerational epigenetic inheritance remains unproven in mammals and may not occur in this system at all [24,26]. Any future signal will be difficult to attribute to radiation specifically, given co-occurring stressors and the confounding effects of admixture, age, disease, parasites and cell composition [13,19,20,23]. Individual movement and true exposure histories are not quantified [5]. Our account of the present state of the population, of the surgical practice of the clinics and of the constraints under which they operate rests in part on the direct experience of two of the authors and on a personal communication from a third researcher working in the zone. The supporting programme data are not yet published; readers should weigh the account accordingly, and the detailed record will be more securely established when that work appears. The magnitude of immigration into the zone since 2022 is unquantified, and no existing baseline captures it. The effects of the armed conflict on dispersal and stress are unknown. The methylation clocks we invoke have known methodological limitations [23]. The post-mortem opportunity described in Section 6.3 is, at present, a proposal rather than an established practice, and its yield would depend on how quickly material could be stabilised under field conditions. The recommendations of Section 6 are, finally, generalised from a single case, and the Chornobyl programme is, in several respects, unrepresentative of the settings to which they are addressed. The figures are schematic, and group sizes are order-of-magnitude estimates from programme reports [2,5].

## 8. Conclusions

The dogs of Chornobyl were the subject of two decisions taken on separate tracks. The first decision, to sterilise, was made in 2017 at the request of the Ukrainian authorities, for reasons of welfare, of zoonotic risk, of the safety of a workforce inside an operating nuclear facility, and of a reproductive rate the environment could not support. It has been carried through to near-complete coverage of the established populations, and we do not dispute it. The second decision, whether this population held scientific value that management would foreclose, was raised but never formally taken, because it arose on the scientific side of a programme whose mandate did not specifically include it. By the time it could have been implemented, the population had substantially ceased to breed.

Two things remain actionable, and both should be done now. The archived material from some 400 already-sampled dogs, including parent–offspring trios and repeat-sampled individuals, should be analysed for methylation and expression: it is the only remaining access to this population as it was, much of it predates both the intervention and the arrivals of the past few years, and it requires no new animals and no change to any policy. The dogs that remain are still alive, still identified and still ageing, so that sampling at veterinary contact, and the retention of tissue when animals die or are euthanised for clinical reasons that have nothing to do with research, would extend the record forward in the only direction sterilisation has not closed. The wider point reaches beyond Chornobyl, where only part of it can still be applied. Free-roaming populations at other contaminated sites are not under population control, and comparable decisions will be taken after future contamination events. The scientific evaluation should be scheduled alongside the management decision, and the material that a sterilisation clinic already removes should be retained, so that a population is not sterilised before anyone has asked what it could have answered. At Chornobyl, the answer to that question might well have been that management should proceed exactly as it did; but this option was never systematically considered.

## Data Availability

No new data were created or analyzed in this study. Data sharing is not applicable to this article.
